# A randomised controlled trial of extended brief intervention for alcohol dependent patients in an acute hospital setting (ADPAC)

**DOI:** 10.1186/1471-2458-11-528

**Published:** 2011-07-04

**Authors:** Lynn Owens, Graham Butcher, Ian Gilmore, Ruwanthi Kolamunnage-Dona, James Oyee, Liz Perkins, Tom Walley, Paula Williamson, Ken Wilson, Munir Pirmohamed

**Affiliations:** 1 The Wolfson Centre for Personalised Medicine, Institute of Translational Medicine, University of Liverpool (1-5 Brownlow Street) Liverpool (L69 3GL) UK; 2 Department of Gastroenterology, Southport & Ormskirk NHS Trust (Town Lane) Southport (PR8 6PN) UK; 3 Dept of Gastroenterology, Royal Liverpool & Broadgreen University Hospital Trust (Prescot Street) Liverpool (L7 8XP) UK; 4 Department of Biostatistics, Faculty of Health & Life Sciences, University of Liverpool, (Brownlow Street) Liverpool (L69 3GS) UK; 5 Department of Health and Community Care Research Unit, University of Liverpool, (Brownlow Hill) Liverpool (L69 3GB) UK; 6 Department of Health Service Research, University of Liverpool (70 Pembrooke Place) Liverpool (L69 3GL) UK; 7 Division of Psychiatry, Institute of Psychology Health and Society, University of Liverpool (Brownlow Street) Liverpool (L69 3GL) UK

## Abstract

**Background:**

Alcohol dependence affects approximately 3% of the English population, and accounts for significant medical and psychiatric morbidity. Only 5.6% of alcohol-dependent individuals ever access specialist treatment and only a small percentage ever seek treatment. As people who are alcohol dependent are more likely to have experienced health problems leading to frequent attendance at acute hospitals it would seem both sensible and practical to ensure that this setting is utilised as a major access point for treatment, and to test the effectiveness of these treatments.

**Methods/Design:**

This is a randomised controlled trial with a primary hypothesis that extended brief interventions (EBI) delivered to alcohol-dependent patients in a hospital setting by an Alcohol Specialist Nurse (ASN) will be effective when compared to usual care in reducing overall alcohol consumption and improving on the standard measures of alcohol dependence. Consecutive patients will be screened for alcohol misuse in the Emergency Department (ED) of a district general hospital. On identification of an alcohol-related problem, following informed written consent, we aim to randomize 130 patients per group. The ASN will discharge to usual clinical care all control group patients, and plan a programme of EBI for treatment group patients. Follow-up interview will be undertaken by a researcher blinded to the intervention at 12 and 24 weeks. The primary outcome measure is level of alcohol dependence as determined by the Severity of Alcohol Dependence Questionnaire (SADQ) score. Secondary outcome measures include; Alcohol Use Disorders Identification Test (AUDIT) score, quantity and frequency of alcohol consumption, health-related quality of life measures, service utilisation, and patient experience. The trial will also allow an assessment of the cost-effectiveness of EBI in an acute hospital setting. In addition, patient experience will be assessed using qualitative methods.

**Discussion:**

This paper presents a protocol for a RCT of EBI delivered to alcohol dependent patients by an ASN within an ED. Importantly; the trial will also seek to understand patients' perceptions and experiences of being part of a RCT and of receiving this form of intervention.

**Trial registration number:**

ISRCTN: ISRCTN78062794

## Background

### Scale of alcohol dependence in acute hospitals

A wide range of harms can ensue from risky drinking behaviour [1] many of which have to be dealt with by general hospitals on a daily basis. Alcohol is a factor in over 40 medical conditions that can lead to hospitalisation, which in 2010/11 accounted for 1.1 million hospital admissions in England, an increase of 12% compared with 2008/09 [2,3]. This figure does not include the 12% of ED attendances that are attributed to alcohol, which increases to 70% at peak times [4]. Additionally, prevalence of alcohol dependence in medical inpatients ranges from 3% to 47% [5,6]. Furthermore, over the past two decades, alcohol-related death rates have doubled [7]. The resulting annual cost to the UK NHS has been estimated at between £2 and £3 billion [8,9]. A rigorous assessment of alcohol-related harm showed that 38% of men and 16% of women (aged 16-64 years) have an alcohol use disorder, equating to approximately to 8.2 million people in England [10]. Of most concern is the estimate that 3-5% of the population in England (1.1 million people) are dependent on alcohol. However, treatment in acute settings would seem to be woefully inadequate, as in most cases alcohol dependent hospitalised patients are given pharmacological detoxification and, when stable, discharged into primary care without further treatment or support. This is a missed opportunity on several levels: early identification and treatment can reduce subsequent individual harm, and may prevent desease pregression, which will ultimatly reduce the overall burden of alcohol to society as a whole and the NHS in particular.

### Evidence for effectiveness of treatment in acute care settings

When considering brief intervention (BI) as a treatment approach for alcohol dependence in an acute care setting, it is important to note that the intensity of intervention does not seem to be a predictor of treatment effectiveness [11-15]. For that reason, brief treatments may be as effective as more intensive treatments in some patient groups. It is therefore noteworthy that, for those patients who are dependent on alcohol, treatments that are brief, timely and pragmatic have received little research focus. Furthermore, the assessment of the effectiveness of treatment is complex; particularly since between 12 to 35% of patients recover with little or no specialist intervention, a phenomenon described as "natural recovery" [16-19]. It has also been established that irrespective of their similarities and differences treatments seem to perform equally well, or badly [20,21].

It is well established that brief treatments are effective for hazardous and harmful drinkers [22] but may also have some efficacy in alcohol-dependent patients [22-30]. However, BI as a treatment option in acute hospitals has yet to be systematically tested in dependent drinkers [31-34]. The ED has been shown to be an appropriate setting in which to identify non-treatment seeking hazardous and harmful drinkers [31,35], and is potentially best placed to deliver effective care based on a BI approach [22,23,25-29,35,36]. However, to date, there has been little focus on the effectiveness of BI in alcohol dependence. A pragmatic non-randomised controlled study (Quaisi-experimental) of BIs by ASNs showed that 30% of alcohol-dependent patients' maintained abstinence for 6 months post treatment [37]. A subsequent study showed that patients treated by the ASN had significantly shorter stay in hospital and a significant reduction in alcohol consumption and dependence at 6 months [38]. Furthermore, a recent retrospective evaluation found that alcohol dependent patients realised as much benefit as non-dependent patients from BIs delivered by an ASN in acute care [35].

Given the scale of the problem, and the lack of choice currently available for this group of patients, it is vital to develop novel models of care which focus on the delivery of effective treatments in the acute care setting where this patient group most frequently presents. Indeed, Saitz proposed that enhanced brief intervention strategies may be effective in decreasing the direct complications associated with unhealthy alcohol use [11,39].

## Methods/Design

### Hypothesis

EBIs delivered to alcohol-dependent patients in a hospital setting by an ASN will be effective when compared to usual care in reducing overall alcohol consumption and improving on the standard measures of alcohol dependence.

### Overall aim of the study

To test the clinical and cost effectiveness of extended BI for individuals with alcohol dependence in an acute hospital setting.

The study objectives are:

1. To determine whether BI reduces overall alcohol consumption.

2. To determine whether BI is effective in the treatment of alcohol dependence.

3. To determine whether BI reduces the length of stay in hospital

4. To determine how patients feel about being approached in an opportunistic manner for screening for alcohol-related problems and subsequently being asked to be part of a research trial.

5. To determine the cost effectiveness of BI for alcohol dependence

The study is a randomized controlled trial. The study has been approved by North West Research Ethics Committee ref: 09/H1005/61, and is fully compliant with the Helsinki declaration 2008.

### Setting

The trial will be conducted within an acute NHS Hospital Trust with an ED based within the North West region of England. In order to minimise the risk of contamination from other alcohol interventions, the setting currently has no protocols for screening or advice within their ED or hospital wards.

### Design

The trial comprises three parts: the first is a test of the clinical effectiveness of the EBI, the second is an assessment of the patient's experience of the intervention using qualitative methods, while the third is an assessment of cost effectiveness of the BI.

### Blinding

The nature of delivery of EBI precludes blinding of subjects. However, the nurses conducting the follow-up assessments at 12 weeks and 6 months remain blinded to treatment allocation. The statistician involved in data analyses will be blinded for treatment allocation.

### Trial inclusion and exclusion criteria

Trial inclusion and exclusion criteria have been selected to ensure that patients are able to understand the importance of adherence to the trial protocols, and are able to provide contact details for follow-up. It is also important to ensure that patients' medical co-morbidity will not necessitate long term hospitalisation, as this will prevent the delivery of EBI as an out-patient. Furthermore, to prevent contamination by other treatments the patient must not have been involved in any alcohol-specific treatment for alcohol dependence in the previous 6 months.

All ED attendees over the age of 17 years, with a score equal to or greater than 16 on the AUDIT screening tool, who live within the local area and are amenable to attend follow-up clinics for interventions, are eligible.

### Trial Recruitment Process (See flow diagram Figure 1)

**Figure 1:**
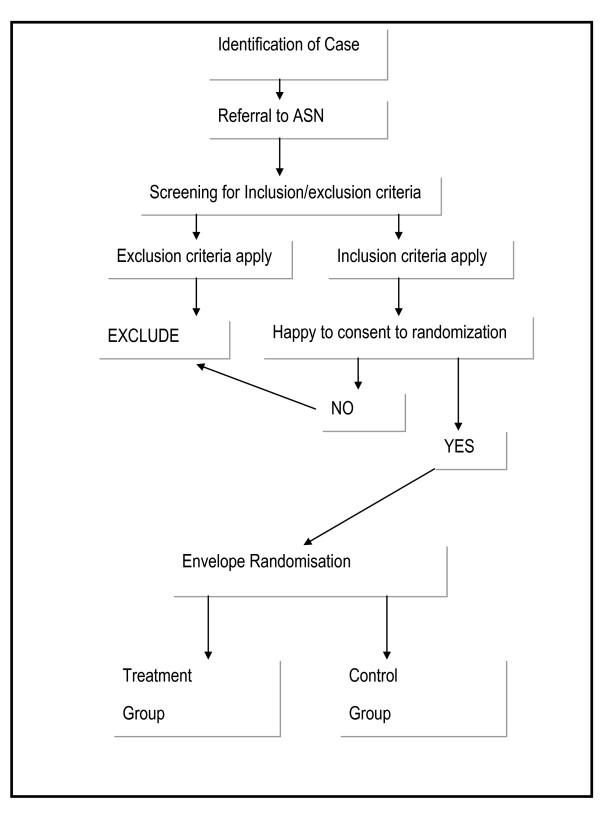
**Trial Recruitment Process**.

Clinicians performing treatment or triage within the ED will utilise their clinical examination to determine the likelihood of the admission being alcohol-related. On identification or suspicion of an alcohol-related problem, the clinician will refer to the ASN for further assessment. The ASN will also visit the ED and Medical Assessment Unit daily to identify cases. The ASN will then screen all patients using the AUDIT screening tool. An AUDIT score of 16 and above will be considered a positive screen. Patients will then be assessed against inclusion/exclusion criteria.

### Trial consent procedure

All eligible patients will be given relevant verbal and written information about the research and asked to consent. Due to the opportunistic nature and design of the study, patients will be asked to provide written consent on initial assessment. This is to ensure that the population recruited is representative of the clinical population attending ED whether aware or unaware of the role of alcohol in their presentation. Patients will be able to withdraw from the study at any point. If reasons for opting out are volunteered by the patient, we will ask consent to use this information. The ASN will be the nurse taking consent from the patient and will discuss with the patient's named nurse if they agree that the patient is physically and mentally fit to give consent to the study. The patient will be provided with a patient information leaflet (PIL) which the nurse will discuss in detail. If the patient gives consent, they will undergo a comprehensive assessment at which the ASN will collect all baseline demographic data, information on co-morbidity, complete all screening tools, and take a full alcohol history. Those patients unwilling to consent to randomisation will be given usual clinical care, and be asked to consider consent for their demographic data to be collected, and will be asked if they wish to take part in the qualitative part of the study. A separate PIL will be used for the qualitative analysis, who will then be interviewed by the qualitative researcher (QR).

### Trial randomization procedure

Following consent, the research nurse will complete the screening and diagnostic tools:

a) Severity of Alcohol Dependence Questionnaire (SADQ),

b) Leeds Dependency Questionnaire (LDQ),

c) Readiness to Change Questionnaire (RCQ), and

d) EuroQoL EQ-5D.

The patient will then be randomized to either the intervention or control group by the research nurse. Patients will be randomized using sequentially numbered opaque sealed envelopes prepared according to a computer-generated randomisation allocation sequence. Block randomisation using randomly varying block sizes (prepared using Stata version 8.2) will ensure equal numbers of patients are recruited into each group. Randomisation services will be provided by an independent statistician at the Clinical Trials Research Centre, University of Liverpool.

### Data collection/management process

#### **Intervention**

All data will be collected prospectivley utilising a Case Report Form (CRF). Data will be collected at baseline and each follow-up point. Data from the CRF which will carry only an anonymised number will be entered directly on a MACRO™ electronic database system. Patient information will be kept on a patient log, which will remain in a locked filing cabinet in a clinical room at the clinical site, alongside the anonymised case report files.

#### **Qualitative**

Interviews will be digitally recorded, anonymised and transcribed by a third party. All digital files will be destroyed within 12 weeks of interview. NVivo, computer assisted qualitative data analysis software that stores and retrieves data will be used.

#### **Economic**

For retreval of hospital utilisation data, researchers will use hospital based electronic recording systems. For GP data, the researchers will visit individual patient GP surgeries and collect data from case files and electronic systems. Ambulance data will be collected electronically. All data will be entered onto the CRF, and will be entered directly in to a MACRO™ electronic database system.

### Primary outcome measures

As in previous research by our team, the primary outcome measure for the study is the change in SADQ score at 6 months post randomisation. The SADQ is a short 20-item questionnaire designed to measure severity of dependence on alcohol [40]. There are five subscales with four items in each: Physical Withdrawal, Affective Withdrawal, Withdrawal Relief Drinking, Alcohol Consumption, and Rapidity of Reinstatement. Each item is scored on a 4-point scale, ranging from "Almost Never" to "Nearly Always" resulting in a corresponding score of 0 to 3. Thus the total maximum score possible is 60 and the minimum is 0.

### Secondary outcome measures

The secondary outcome measures are:

1. A change in AUDIT score from baseline at 12 weeks and at 6 months post randomisation. Although AUDIT is a screening test for alcohol use disorders [41], in common with other studies, we will in this context use it as both an outcome measure and a pre-screen to establish presence of alcohol dependence. The sum of the items scored has been used in several studies to indicate dependence and therefore will allow for comparability with other trial baseline and outcome measures [10].

2. Measure of alcohol dependence utilising the Leeds Dependency Questionnaire (LDQ) [42] at baseline, 12 weeks and 6 months post randomisation. The LDQ is a 10-item questionnaire designed to measure dependence upon a variety of substances including alcohol. The questionnaire includes 1 item from 10 markers that define dependence; these include pre-occupation, salience of alcohol, compulsion to start, planning around alcohol consumption, maximize effect, narrowing of use of repertoire, compulsion to continue, primacy of effect, constant state and cognitive set. Each item is scored 0-1-2-3, giving a maximum of 30 with higher score indicating greater degree of dependence.

3. Quantity and frequency of alcohol consumption in UK units per drinking day at baseline, 12 weeks and 6 months post randomisation.

4. Readiness to Change Questionnaire (RCQ) at baseline, 12 weeks and 6 months post randomisation. This is an important measure for non-treatment seeking populations as it has been demonstrated that treatment-seeking is an indication of treatment readiness, which has been seen as important in achieving treatment outcomes [20]. Using this measure will help us to investigate if treatment readiness does indeed have any bearing on treatment outcome in the population and setting.

5. Number of ED attendances 6 months pre and 6 months post treatment/control.

6. Hospital length of stay sum days in hospital 6 months pre and 6 months post randomisation.

7. Number of hospital admissions 6 months pre and 6 months post randomisation.

8. Length of stay for initial treatment in days.

9. Biochemical indicators of co-morbid conditions including Gamma-glutamyl transferase (GGT), Alanine aminotransferase (ALT) and mean corpuscular volume (MCV), when available will be extracted from a recent laboratory records; this is defined as the last available value prior to randomization.

### Sample Size calculation

Based on a previous study by ourselves on dependent drinkers receiving interventions from an ASN, it is expected that 55% of such patients will display a fall in SADQ score between baseline and 12 weeks' follow up [38]. Another confounder in this area of research is the phenomenon of natural recovery (NR). The NR rate over this time period expected in the control group is expected to be no more than 25% (the literature ranges from 12% with a treatment population [40] up to 35% within a general lifetime population [43,44]). In order to detect this difference between the groups (55% vs. 25%) with 90% power at the 5% significance level, approximately 65 patients are required in each group. In order to allow for an estimated 50% drop out rate (observed in previous studies in similar patients), 130 patients will be recruited per group. It is estimated that at least 5 eligible patients will be recruited per week, and thus this target should be achieved within the 12 month recruitment period.

A fall in SADQ at 6 months is be the primary outcome. As there were few studies that report SADQ scores at baseline and follow-up, the revised power calculation was based on the our own studies that 22% vs 53% fall in SADQ between control and Intervention arms at 6 months. In order to detect this difference between the groups (55% vs. 25%) with 90% power at the 5% significance level, the required size is 103 (51 per group), and 206 (103 per group) once adjusted for 50% dropout.

### Part A-Treatment group

Patients will receive an initial assessment and EBI from the ASN. The EBI can best be described as a motivational approach to helping individuals change their drinking behaviour. The intervention builds upon methods utilised to deliver BIs. However, as the intervention is delivered for approximately 20 minutes on up to six occasions the term "brief" is somewhat anomalous. We will therefore refer to this mode of delivery as an "extended brief intervention" (EBI). The intervention will be delivered utilising the FRAMES approach [25], and will be protocol driven with regular clinical supervision and observation to maximise intervention integrity. Each intervention will be documented and timed. The most important element of this model is the exploration of patients' perceptions as to the link between their alcohol consumption and presentation. The FRAMES [45] approach consists of the following:

FEEDBACK about personal risk or impairment;

RESPONSIBILITY: emphasis on personal responsibility for change;

ADVICE to cut down or abstain if indicated because of severe dependence or harm;

MENU of alternative options for changing drinking pattern and, jointly with the patient, setting a target;

EMPHATIC INTERVIEWING: listening reflectively without cajoling or confronting; exploring with patients the reasons for change as they see their situation;

SELF EFFICACY: an interviewing style that enhances peoples' belief in their ability to change.

A maximum of six treatment sessions, lasting 20 minutes each, will take place within the 12 week treatment period (2 hours in total). Follow-up visits will be scheduled 12 weeks and 6 months following recruitment into the study, and will be performed in the outpatients department, at home or by telephone. The assessor will be blinded to treatment allocation.

### Control group

The control group will receive the same assessments as the intervention group but they will not receive the EBI. They will receive normal clinical care, which may or may not include referral to a specialist alcohol service. Follow up visits will be arranged 12 weeks and 6 months following recruitment into the study, either in outpatients, at home or by telephone by a nurse researcher blinded to treatment group.

### Patient experience

The qualitative researcher (QR) conducting face-to-face interviews will receive contact information for patients consenting to this part of the trial. The QR will contact the patient to arrange a time and place for the interview. The patient experience part of the trial adopts a qualitative design and will focus on gaining the patient's views on three main features of the study: a) of being screened for alcohol-related problems, b) of being invited to take part in a treatment programme, c) of being randomized. It is designed to capture the patient's experience and more particularly the opportunistic nature by which they will have their drinking assessed and of their participation in the RCT. In-depth face to face interviews will be used to collect data using a topic guide. The topic guide is designed to elicit information about the following:

▪ The experience of the opportunistic nature of the screening.

▪ The experience of being offered and receiving a BI.

▪ The experience of being identified as alcohol dependent without being included in the intervention arm of the study.

The identification of themes and patterns in the interview data, using such techniques as constant comparative and deviant case analysis, will be the predominant method of organising, coding and categorizing the data.

### Economic Evaluation

For the economic analysis, Euroqol EQ 5D [46] will be utilised. EQ-5D is a standardised instrument for use as a measure of health outcome. The first part of the EQ-5D has 3-levels consisting of 5-dimensions including mobility, self care, usual activities, pain/discomfort, and anxiety/depression. The second part is a visual analogue scale (VAS) used by the patient to subjectively measure on a point interval scale their health status. Worst imaginable health state is scored as 0 at the bottom of the scale, and best imaginable health state is scored as 100 at the top.

The major part of the economic analysis will be a simple comparison of costs in the two arms, measuring the costs of providing the services against any differences in NHS costs between the two arms which might occur as a result of decreased use of all other services (for example, hospital reattendances, accidents, GP visits etc) regardless of their relationship to the abuse of alcohol. The perspective will be limited to that of the NHS. The time frame will be over the six months follow-up time of the study. The costs of the intervention will be as identified in the study. The costs of the use of NHS services (A/E or GP attendances, cost per bed day as an inpatient) will be taken from standard NHS costs published by the [47]. The total costs of NHS care for each patient will be based on these costs and on the amount of service utilisation data collected as outlined above. It may be necessary to adjust the analysis if there are differences in baseline SADQ or in comorbidities.

A separate analysis of NHS costs specifically for alcohol related services will need to be undertaken as a reduction in NHS service use may only be seen in alcohol-related areas, especially in patients with severe comorbidities. Whether a particular NHS intervention is related to alcohol or to another cause will require judgment, which will be undertaken by two clinical assessors who are blind to patient allocation.

If the costs (especially total costs but also alcohol only related costs, provided that non alcohol related costs are equal) in the intervention arm are less than in the non-intervention arm, then the economic case for the intervention is made. If the costs in the intervention arm are greater than in the non-intervention arm, it is necessary to undertake a cost effectiveness study, with the same time frame and perspective.

### Follow Up Strategy

This population can be difficult to follow-up and loss to follow-up in this setting has been reported as high as 48% [38]. Therefore, the research nurse will take an address and telephone details for the patient or relative/carer. The patient will also be asked to give permission for the research team to contact their General Practitioner (GP) to obtain additional data. To facilitate ease of access for treatment and follow-up, patients will be offered a range of follow-up options either in the hospital, on the telephone, or at home. Two weeks prior to the follow-up appointment, the patient or other named contact will be contacted to determine if they will be attending their appointment. The day before their appointment, patients will be contacted to remind them that their follow-up appointment is due the next day. If the patient is unable to be contacted, they will be sent a letter asking them to contact the team in order to arrange follow-up. If the patient is unable to be contacted via telephone or fails to respond to a letter for the 6 month follow-up appointment, the research nurse will visit the patients home to determine a) if the patient still resides at that address, and b) if the patient is still willing to undertake a follow-up appointment. If the patient cannot be contacted by the team at this stage, they will be said to be lost to follow-up.

### Ethical Considerations

Each group will receive normal clinical care in their respective groups. No patient group will be disadvantaged as clinical care in these patients varies, and we do not know which the most effective treatment is.

### Planned Analysis

An intention to treat analysis (ITT) will be performed on all analyses. This will include all patients assigned to the two groups; EBI or standard care as randomised, irrespective of the patient's compliance with the study protocol or the actual study intervention received. A per protocol analysis will also be performed. The per protocol analysis will comprise all patients in the ITT population who have met all the following major inclusion criteria and who do not have any major protocol violation. A sensitivity analysis will be applied for any missing primary outcome data.

To ensure the appropriateness of the event rates used in this sample size calculation, an internal pilot is planned after 160 patients will have been randomised. The two main reasons for performing the internal pilot are:

• Evaluation of overall proportion of patients displaying a fall in SADQ score to compare the estimate used in the original sample size calculation.

• Monitoring study recruitment and missing data for all primary and secondary outcomes.

The only outcome data that will be analysed within the internal pilot analyses is the proportion of patients displaying a fall in the primary outcome of the study (SADQ) between baseline and 12 weeks' follow-up. The results will be presented as numbers and percentages and the proportion of patients showing a fall in SADQ used in the original sample size calculation will also be checked by using the estimates that are obtained from the internal pilot analysis. Outcomes showing unexpected missing information will be highlighted and discussed with members of the Independent Trial Steering Committee (ITSC). If sample size recalculation suggests need for more patients to preserve power to detect treatment effect, then on the advice of the ITSC, we will aim to increase recruitment and consider implications for funding and existing resources.

### Analysis of primary outcomes

The primary endpoint is a fall in SADQ at 6 months post randomisation. Test scores will be summarised using descriptive statistics, means with 95% confidence intervals (or medians with inter-quartile ranges if non-normally distributed) at baseline, and 6 months. The hypothesis of no difference between the two treatment arms at 6 months (separate analyses) will be tested using analysis of covariance (ANCOVA), controlling for baseline measurements. A p-value of 0.05 (5% level) will be used to declare statistical significance and 95% confidence intervals of the estimated effects will be reported. Time by treatment interaction will be assessed. The primary analysis using ANCOVA will not adjust for any missing data. However, reasons for missing outcome data will be reported and a sensitivity analysis will be undertaken. The assumptions that are made when using ANCOVA (i.e. normality of scores at treatment levels, homogeneity of variance, homogeneity of regression slopes, linear regression) will be assessed. If unequal variances, nonlinearity and/or non-parallel slopes are present, a suitable transformation of scores will be employed to improve the linearity and to promote equality of the variances.

### Analysis of secondary outcomes

The AUDIT and AUDIT-C, LDQ and RCQ scores will be summarised using descriptive statistics: means with 95% confidence intervals (or medians with inter-quartile ranges if non-normally distributed) at baseline, 12 weeks and 6 months. Change from baseline at 12 weeks and at 6 months post treatment will be presented. The hypothesis of no difference between the two treatment arms at 12 weeks and 6 months (separate analyses) will be tested using analysis of covariance (ANCOVA), controlling for baseline measurements. A p-value of 0.05 (5% level) will be used to declare statistical significance and 95% confidence intervals of the estimated effects will be reported. Time by treatment interaction will be assessed. This analysis using ANCOVA will not adjust for any missing data. However, reasons for missing outcome data will be reported and a sensitivity analysis will be undertaken Time by treatment interaction will be assessed. The mean (standard deviation) or median (inter-quartile range) of quantity and frequency of alcohol consumed in UK units per drinking day will be computed depending on whether it is skewed or not, and compared across treatment groups at 12 weeks and at 6 months post treatment using a t-test or Mann Whitney U test. The number of ED attendances is also similarly analysed. Summaries of length of stay in hospital will be presented as means (standard deviations) or medians (inter-quartile ranges) depending on whether it is normally distributed or not, and compared across treatment groups. Laboratory parameters will be summarized using means, standard deviations, confidence intervals, and ranges. Additional summaries of the data included plots of mean (±SE) values over time and scatter plots of pre- vs. post-treatment values. Change from baseline and at 12 weeks and at 6 months post treatment will be presented. A formal test of a treatment-covariate interaction will be conducted by including the interaction term in a regression model. Exploratory analysis will be conducted as to the impact on any treatment effect of other factors such as gender or age. A p-value of 0.05 (5% level) will be used to declare statistical significance and 95% confidence intervals of the estimated effects will be reported.

### Missing data and missing questionnaire items

To investigate how sensitive the results of the primary and secondary analysis are to missing data a number of strategies will be used including joint modelling as well as imputing values for missing longitudinal scores at 12 weeks and 6 months. The results of the sensitivity analyses will be compared to assess the relative effect of missing data on the conclusions of the primary and secondary analysis.

For a scale which is based upon a number of items, of which one or more is missing, to investigate how sensitive the results of the primary analysis are to missing data a number of strategies will be used including a simple imputation where we will estimate the scale score for the missing items from the mean of those items which are available and this mean is then used to replace the two missing values. Missing scale scores will also be imputed with worst-case value, with best-case value and model-based imputation.

### Analysis of patient experience

The qualitative study adopts an approach based on the analytical principles of Grounded Theory. Themes and patterns in the data are identified using constant comparative techniques and data are organised into codes and categories to enable theory to be developed at a substantive level. While the constant comparative method is associated with the principles of grounded theory, comparison represents a central analytic process in inductive qualitative analysis that seeks to derive concepts from data. In analysis the researcher is as interested in 'negative', 'deviant' or 'anomalous' evidence as the identification of patterns of response. In this sort of analysis divergence provides an important way of informing and modifying emergent conceptualisations and explanation.

### Analysis of Health Economic measures

The economic analysis is designed to determine cost effectiveness for delivery of the clinical intervention protocol. This will include analysis of post intervention healthcare utilisation, and standard measures of changes in health status.

## Discussion

This paper presents a protocol for a RCT of EBI delivered to alcohol dependent patients by an ASN within an ED. Importantly; the trial will also seek to understand patients' perceptions and experiences of being part of a RCT and of receiving this form of intervention. Determining the clinical and cost effectiveness of the intervention will help inform service design and planning.

## Abbreviations used

(ASN): Alcohol Specialist Nurse; (AUDIT): Alcohol Use Disorders Identification Test; (AUDIT - C): Alcohol Use Disorders Identification Test - Community; (BI): Brief Intervention; (CRF): Case Report Form; (DH): The Department of Health; (EBI): Extended Brief Interventions; (ED): Emergency Department; (GP): General Practitioner; (ITT): Intention to treat; (LDQ): Leeds Dependence Questionnaire; (NR): Natural recovery; (PEQ): Patient Experience Questionnaire; (QR): Qualitative Researcher; (RCT): Randomised Control Trial; (RCQ): Readiness to Change Questionnaire; (SADQ): Severity of Alcohol Dependence Questionnaire;

## Competing interests

The authors declare that they have no competing interests.

## Authors' contributions

All of the authors contributed to the design and development of the trial protocol. LO wrote the first draft of the paper. MP, IG, KW, TW, RK, JO, PW, LP, GB, commented and contributed to successive drafts. All authors read and approved the final manuscript.

## Pre-publication history

The pre-publication history for this paper can be accessed here:

http://www.biomedcentral.com/1471-2458/11/528/prepub
